# Uptake of and adherence to oral pre-exposure prophylaxis among adolescent girls and young women at high risk of HIV-infection in Kampala, Uganda: A qualitative study of experiences, facilitators and barriers

**DOI:** 10.1186/s12905-022-02018-z

**Published:** 2022-11-10

**Authors:** Ivy Kayesu, Yunia Mayanja, Catherine Nakirijja, Yvonne Wangũi Machira, Matt Price, Janet Seeley, Godfrey Siu

**Affiliations:** 1 Medical Research Council, Uganda Virus Research Institute and London School of Hygiene and Tropical Medicine Uganda Research Unit, P.O. Box 49, Entebbe, Uganda; 2 International AIDS Vaccine Initiative (IAVI), 125 Broad Street, 9th Floor, 10004 New York, NY USA; 3 Department of Epidemiology and Biostatistics, University of California, San Francisco (UCSF), 550 16th St, 94158 San Francisco, CA USA; 4 Global Health and Development Department, London School of Hygiene and Tropical Medicine, 15-17 Tavistock Place, WC1H 9SH London, UK; 5 Child Health and Development Centre, Makerere University, Mulago Hill Road, Kampala, Uganda

**Keywords:** Qualitative methods, Pre-Exposure Prophylaxis, Adolescent Girls and Young Women, Socio-Ecological Model, HIV prevention, Sub-Saharan Africa

## Abstract

**Background:**

There is limited information on factors that influence oral pre-exposure prophylaxis (PrEP) uptake and adherence among adolescent girls and young women (AGYW). We conducted a qualitative methods study to explore experiences, facilitators and barriers of PrEP uptake and adherence to PrEP among AGYW at risk of Human Immunodeficiency Virus (HIV) infection in Kampala, Uganda.

**Methods:**

This study was nested in a prospective cohort study that offered daily oral PrEP to AGYW. Between April 2019 and October 2020 we conducted in-depth interviews with 26 AGYW aged 14–24 years who had been offered or had been using PrEP for at least 6 months, including PrEP adherers (8), non-adherers (8) and those who had declined PrEP (10). After 12 months, follow-up interviews were conducted with 12 AGYW who had adhered to PrEP and those who had dropped it. Thematic analysis was conducted and data were further examined and categorized into the 5 constructs of the Socio-Ecological Model (SEM).

**Results:**

PrEP uptake and adherence were facilitated by factors including: perceptions that one’s own or partner’s sexual behaviour was high risk, a negative attitude towards condoms, social support and wanting to maintain a negative HIV status after receiving a negative HIV test result. Good adherence to PrEP was enabled by effective counselling, support tools such as alarms and phone reminders and incentives like free treatment for STIs and other illnesses during study visits. Barriers to uptake included: anxiety about the pill burden, perceptions of being too young for PrEP and fear of being labelled `prostitute’ or `HIV positive’. Poor adherence was attributed to doubt over the efficacy of PrEP as a result of beliefs that because HIV was incurable, no medicine could prevent it. Alcohol use, side effects experienced, and mobility all had a negative impact on adherence. The majority of PrEP users reported feeling safe as a result of using PrEP which had both good and negative implications on their sexual behaviour, specifically the number of sexual partners and condom use.

**Conclusion:**

Addressing community misconceptions to maximize uptake of PrEP among AGYW is important. Targeted education messages, and counselling to address misconceptions in ways that capture the attention of AGYW in communities are required.

## Background

Oral PrEP provides hope for a region that still accounts for the biggest proportion of new Human Immunodeficiency Virus (HIV) infections globally, and where adolescent girls and young women (AGYW) continue to contribute the highest proportion to new infections [1]. Oral PrEP is being scaled up not only to key populations, but also to other populations in sub-Saharan Africa (SSA). South Africa, Lesotho, Zambia and Kenya among other countries have national programs targeting other populations including adolescent girls and young women (AGYW), sero-discordant couples and the general population [2]. In 2018, the Ugandan government issued technical guidance recommending Pre-Exposure Prophylaxis (PrEP) for HIV negative persons at substantial risk of HIV acquisition. These guidelines stated that PrEP would be available at a few funded accredited sites which offer Anti-Retroviral Therapy (ART) and it would not be offered in public health facilities [3]. According to an online newspaper article, there were between 9,500 and 10,000 PrEP users in Uganda by the end of 2019 and these were primarily HIV discordant couples and the most at-risk populations like as sex workers. PrEP was reported to be mostly accessed through demonstration sites and facilities accredited to provide PrEP [4]. More recently in 2022, PrEP is said to be available in 260 facilities in Uganda and more than 175,000 people have started using PrEP [5].

In SSA, AGYW aged 15–24 years represent 10% of the total population, but account for about 25% of all new HIV infections [6]. A study carried out among fishing communities in Rakai, Uganda found that HIV prevalence among 15–24 year olds was 19.7% and that HIV prevalence rates are higher among females and yet uptake of HIV prevention services is low [7]. Ethical restrictions on age of participation has hindered and led to the exclusion of adolescents in Uganda, especially those from key populations such as female sex workers (FSWs) from participating in HIV prevention research [8–10] and yet AGYW engaged in sex work are considered one of the most at risk populations in countries with high HIV prevalence and high fertility rates such as Uganda [11]. Additionally, studies from multiple regions representing different geographical and cultural characteristics consistently show evidence that up to 40% of FSWs become sex workers as adolescents [12–14]. Young people in Uganda who engage in high risk sex are up to 7 times more likely to get infected with HIV that other young people [15]. Few studies have followed up cohorts of adolescents and young people in order to inform HIV prevention interventions for this group and yet AGYW are at a high risk of HIV infection and there is need for this group to be involved in HIV prevention research.

Initial reports from PrEP implementation projects show that free access to PrEP, access to support services, such as regular HIV testing, sexual health care/monitoring, and access to one-on-one counselling, are among the most critical facilitators of PrEP uptake [16]. However, low risk perception [17, 18] and limited awareness [19] are important barriers to uptake. Even among those who initially express interest, long term adherence can remain poor [20]. PrEP adherence is reported to be affected by age (< 25years) [21, 22], side effects, fear of stigma associated with use of anti-retroviral (ARV) drugs and negative attitudes from health workers [23, 24].

We explored experiences with PrEP and facilitators and barriers to PrEP uptake and adherence and among volunteers who took PrEP (adherers and non-adherers) and those who declined PrEP, in a cohort of 14-24-year-old AGYW at risk of HIV infection in Kampala, Uganda.

## Methods

### Study setting

We used qualitative methods to gather information on experiences of young people who were enrolled in the “**I**nterventions for HIV **P**revention among **Ad**olescents and Young Women” (IPAD) study. It was a 24-month prospective cohort study that enrolled 285 HIV seronegative AGYW who frequently reported transactional sex. Field workers recruited AGYW from sex work locations, bars, lodges and urban slums with a high concentration of young people and where alcohol and drug use were common. The cohort was based at the Good Health for Women Project (GHWP) clinic in southern Kampala, Uganda and ran from January 2019 to December 2020. The GHWP clinic offered HIV prevention, care and treatment services, and sexual reproductive health services to women at high-risk of HIV infection including female sex workers. IPAD participants were prescribed a once-daily PrEP regimen of Tenofovir/ Lamivudine (TDF/3TC).

### Study population and sampling

Between April 2019 and October 2020, we purposively selected 26 of a planned 30 participants (10% of sample size) from the IPAD cohort. The IPAD cohort enrolled participants aged 14–24 years; those below 18 years were enrolled if identified as emancipated or mature minors who can consent to participate in research as per the 2016 guidelines of the Uganda National Council for Science and Technology (UNCST) [25]. The selection of the final four participants was prevented by COVID-19-related travel restrictions which came into force in March 2020. At the time of recruitment, IPAD participants had been using PrEP for a period of at least 6 months and were selected according to 3 categories; adherers (eight), non-adherers (eight) and those who declined PrEP (ten). Efforts were made to have a cross-section of ages represented in this sample. To help AGYW make an informed decision on whether to join the IPAD cohort study and to support their adherence, they received group education, covering what PrEP was and its purpose. The baseline interviews were conducted at 6 months in the cohort and follow up interviews were conducted at 12 months in the cohort (i.e., 6 months after the baseline interview) among those who took PrEP and were divided into two categories: those who reported adhering to oral PrEP (seven), and those who were not adhering (five). Adherers comprised of participants who consistently had monthly self-reports and pill counts showing optimal adherence (score ≥ 90%) over the period they were taking oral PrEP, while non-adherers had monthly reports and pill counts showing sub-optimal adherence (< 90%) or started using PrEP and stopped even when they continued to be at risk of HIV infection.

### Data collection and analysis

Participants were invited for in-depth interviews (IDIs) through phone calls and by face-to-face interaction during their IPAD study visits. A trained social science research assistant, fluent in Luganda, the local language, conducted a total of 38 baseline and follow-up interviews in a private room at the GHWP clinic. An IDI topic guide was used which covered demographic characteristics, knowledge and awareness about oral PrEP, HIV risk perception, experiences with oral PrEP, and facilitators and barriers to PrEP uptake and adherence. Interviews lasted 40–60 min, and were audio recorded. The recordings were transcribed verbatim and translated into English, and transcripts cross-checked against the audio recordings to ensure accuracy.

Thematic analysis was used to analyse the translated data. The data were manually coded by 2 trained research assistants and eight out of the 38 transcripts were initially coded and compared to ensure consistency. A preliminary codebook was developed and refined by three experienced researchers leading to the merging and rephrasing of codes and the list of codes was cut down from 52 to 27 codes. Codes were derived both deductively and inductively based on research objectives and based on patterns or findings emerging in the data set. Excel tables were used to categorize data according to the research objectives and identify patterns. In this paper we report the overall findings from the IDIs. All quotes in the results are from the baseline interviews, except where noted as from the follow up, month 12 interviews.

## Theoretical framework

Health outcomes are increasingly being recognized in literature as being less influenced by individual behaviour and more by the wider environments in which people live [26, 27]. The socio-ecological model (SEM) illustrates 5 interdependent levels of influence which link individual behaviour, environments with which individuals interact and health outcomes [28, 29]. The SEM is useful in understanding the factors that influence health behaviour including HIV prevention [30, 31]. The 5 levels of the SEM are described as; (i) Individual factors such as knowledge, attitudes, and product attributes; (ii) Interpersonal factors describing the influence of social interactions on health decisions; (iii) Structural factors such as access to services; (iv) Institutional factors such as support tools provided and health provider aspects and (v) Community factors such as community perceptions and social norms. We use this construct to examine the impact of PrEP use on normative behaviour among AGYW, who frequently reported paid sex, and examine the factors that facilitate and impede uptake and adherence to PrEP among this population.

We used the SEM framework as a guide to address how different factors influence uptake of PrEP across multiple levels. We incorporated the framework into our analysis following the initial analysis. Similar and interrelated codes were grouped into clusters and reorganized under the 5 constructs of the SEM framework. This allowed us to further examine how the different factors identified in the data were linked to the theories and constructs of the SEM framework.

## Findings

The 26 AGYW who participated were aged between 14 and 24 years, 16 of whom were aged 20–24 years old. Two in three had secondary education, 18 were single, never married and 15 had at least one biological child. Only 6 reported sex work as their main job although many reported engaging in paid sex. Other jobs reported included working in the hospitality or entertainment sector, including bars and lodging facilities, which often also serve as brothels in the study setting (Table 1).

**Table 1:** Demographic and Other Characteristics of AGYW Invited for In-Depth Interviews (N = 26)

Characteristics	Categories	No. of Participants
Age range	14–19	10
	20–24	16
Marital status	Married	6
	Single (never married)	18
	Single (separated/ divorced)	2
Highest education level	Primary	8
	Secondary	16
	Tertiary	2
Employment type	Jobless	9
	Sex work	6
	Other (hospitality, entertainment, cleaning)	11
PrEP awareness before joining study	Aware of PrEP	4
	Unaware of PrEP	22
Number of biological children	One or more	15
	None	11
PrEP use after enrolment	PrEP users (adherers)	8
	PrEP users (non-adherers)	8
	Non- PrEP users	10
Living situation	Living independently (alone, with a spouse, with a sibling)	19
	Living with a guardian (parent or relative)	7

## Factors influencing uptake and adherence to PrEP

**Fig. 1:**
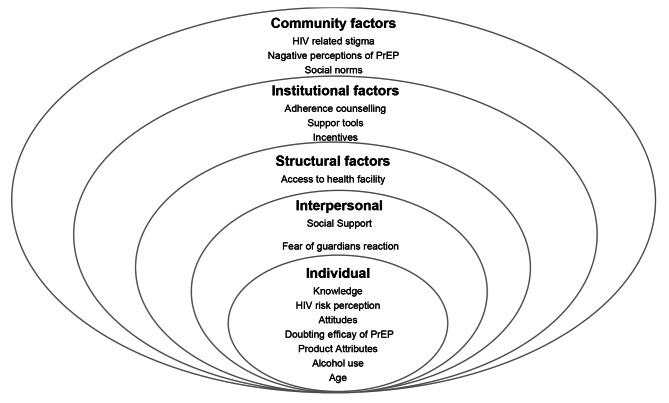
Factors influencing PrEP uptake and adherence among AGYW at each level of the socio-ecological model

Participants described a range of important factors that influenced their uptake and adherence to PrEP. Based on the ecological framework, we present these factors as individual, interpersonal, structural, institutional, and community factors. Figure 1 shows the factors influencing PrEP uptake and adherence among AGYW at each level of the socio-ecological model.

## Individual factors

Factors which affected uptake of and adherence to PrEP at individual level included: knowledge of PrEP, HIV risk perception, new found appreciation for one’s health, age, attitude towards condoms, doubting efficacy of PrEP, alcohol use, product attributes.

### Knowledge of oral PrEP

We asked participants if they knew about oral PrEP before joining the study and if so, what their source of information was. We found that before joining the IPAD cohort, there was very low awareness and knowledge about PrEP among this high-risk group. Most had never heard about PrEP at all, only learning about it for the first time at the study site. A 24-year-old non-adherent woman working in entertainment, primary level educated and single, remarked: “I first heard about it from here [GHWP study site]. I had never heard about it”.

Out of the four participants who were aware of PrEP before joining the study, three reported that they had come across the word ‘PrEP’ in health facilities or heard it from peers, e.g., as was shared by a 22-year-old adherent participant who is a mother of one, divorced, secondary level educated and jobless: “It’s just that you can go to a health facility and you see the word [PrEP], but since you are not using it you don’t really concentrate on it”.

In addition to peers and health facilities they attended, the other source of information mentioned was the television.

I first heard about it that side where I stay. There was a program on TV and they were saying that “do you know that a pill which prevents HIV has been released. And there are also other methods which they are researching about.” They were discussing it but I had never seen it (24-year-old PrEP user, primary school educated, married and mother of one and jobless).

While conducting IDIs in the 6 months after enrolment, we explored their understanding and perceptions of oral PrEP after group education, and we found that regardless of age and adherence levels, interviewees had a good understanding of oral PrEP with most of them able to provide elaborate descriptions of what PrEP is, its purpose and how it is used, suggesting a positive impact of the health talks. A 22-year-old PrEP adherer (single, secondary school educated and working at a solar power distribution company) explained:

PrEP is a drug which is swallowed by someone who doesn’t have HIV and it helps them to prevent getting infected with HIV; If you have decided let’s say 7am, that is the exact time when you are supposed to swallow and you are supposed to swallow 1 pill at that exact time without changing the time.

Similar knowledge was demonstrated by a primary level educated, 24-year-old non-adherent, divorced and jobless PrEP user:

PrEP is a drug which prevents getting HIV when you have swallowed it properly; they told us that you are supposed to swallow one pill every day at the same time and that when you miss a dose you don’t swallow two pills. If you have missed for not more than 12 hours, there you can swallow.

### HIV risk perception

Many AGYW reported accepting to use PrEP and adhering to it because they felt they were at a high risk of contracting HIV. However, they appeared to distinguish the influence of their own risky behaviour from the risk resulting from their partner’s sexual behaviour. Some AGYW attributed their decision to accept PrEP to their involvement in sex work and others attributed adherence to mistrust of their regular sexual partner. For example, in discussing why they accepted to use PrEP, two participants explicitly mentioned their involvement in sex work:

I accepted because of the work I am involved in. And when they explained to me I saw that it [PrEP] can help me. And that made me accept it (23-year-old PrEP user, adherer, secondary school educated, single, mother of 1, sex worker).

Being at risk of getting HIV-that is the reason why I have to use it. Because of this work that we do at night, a man can even rape you on your way back home. You don’t even know your lover’s movements. Even he can bring you that sickness. That is why I decided to use PrEP (19-year-old PrEP user, non-adherer, secondary school educated, single, and mother of 2, snack vendor and sex worker).

### New-found appreciation for one’s health

Another frequently discussed reason for accepting to take PrEP was the new-found appreciation for one’s health. Many AGYW involved in sex work expected to be HIV positive, but those who took the HIV test during their study visits in the IPAD cohort study and discovered that they were HIV negative felt a great sense of relief. The knowledge of their HIV status prompted the desire to protect themselves to remain HIV negative. “I don’t miss [to take PrEP pills] because I love my life and I want to be healthy so that I can work.” (22-year-old PrEP user, adherer, secondary school educated, single, works in salon).

Some participants described having known and appreciated the benefits of PrEP and declared their commitment to use PrEP until the end of the study.

I don’t want to miss [taking pills] because it helps me. So if I am to miss, how will I be protecting myself? That will mean that I don’t even understand what I am doing to protect myself. (Follow-up IDI, 21-year-old PrEP user, adherer, secondary school educated, single and mother of 1, library cleaner and sex worker).

### Age

Younger participants (≤ 19 years) attributed their lack of interest in PrEP to their age. Perceptions that younger girls should not be having sex caused a fear among those AGYW of being judged by people in their communities for being sexually active. This fear therefore discouraged uptake.

In the future I will use them. Because you may find that being young is what is disturbing me [Preventing me from starting PrEP]. (17-year-old non- PrEP user, secondary school educated, single and living with mother, considered emancipated minor because she works to cater for hers and her mother’s livelihood).

Okay, people can say that “You are still young. Why do you want to use those pills?” They can say that “You haven’t even started things to do with having sex” (19-year-old non-PrEP user, secondary school educated, single, and jobless).

### Attitude towards condoms

Dislike for condoms motivated PrEP uptake since PrEP was seen as a better HIV prevention method than condoms which were disliked for their perceived shortcomings, such as risk of getting stuck inside a woman and maintaining sexual partners who do not favour condom use. There were several examples of this concern, but the fears of this participant are illustrative:

The truth is that I am not so interested in using those things [condoms]. Because in our area there is a girl- she is a woman in fact, married. Her husband I think had gotten tired and he left a condom inside her. They looked for the condom and failed to find it. After 3 days the husband told her that he left it inside her and that he was tired of her. So if he does that to you and leaves it inside you, you just have to leave him. Until the woman went for a clinical procedure and they removed it from her. So I think that someone can do that to you and leave it inside you. So that caused me to say that let me swallow those pills (19-year-old PrEP user, adherer, secondary school educated, single, and works in solar power agency).

Another example is provided by a participant who favoured using PrEP because she feared that suggesting condoms with her regular partner would raise suspicions of infidelity.

I use them [condoms] with someone whom I don’t trust. But if you know someone, you don’t consider it so much because if it is your boyfriend and you tell him to use a condom, he can ask you that “are you suspicious of me and why are you suspicious after all this time that I have been with you?” (22-year-old PrEP user, adherer, secondary school educated, single mother of 1, sex worker).

### Doubting efficacy of PrEP

Doubting the efficacy of PrEP in preventing HIV negatively impacted adherence for some participants. Participants also reported community members expressing doubts about the efficacy of PrEP. One participant talked about how she used to miss doses due to doubt.

Because I had never heard about it [PrEP] and I had never even seen it. In fact, that first tin, I only swallowed half of it. I didn’t finish it. I only swallowed half and said that these things don’t work. Sometimes even when you go to church there are times when you say that God does not exist. But when you stay and have faith you see that he is working for you. (24-year-old PrEP user, non-adherer, secondary school educated, single, jobless).

Perhaps the strongest source of doubt and discouragement came from the reported widespread belief in the community that since HIV was an incurable disease, then no medicine could prevent its spread, as reported by these participants:

They say that there is no drug which can prevent HIV. It [HIV] has been here since a long time ago (20-year-old, non-adherer, tertiary level educated, single, works in catering service).

Most people dispute it [PrEP]. Most of them say that it is not possible […] I think that the reason why they think like that is, since they say that HIV doesn’t get cured, they may think that there is nothing like that which can prevent it (19-year-old PrEP user, adherer, secondary school educated, single, works in solar power agency).

### Alcohol use

Alcohol use reportedly interrupted the pill taking schedules of some participants and caused them to miss doses. A sex worker in the adherer group reported missing a dose one day due to over sleeping and missing her time. For another non-adherer, she reported having difficulty adhering during the first month because of alcohol use.

In the first month I used to sleep and forget to swallow. Because when you drink, sometimes in the morning you have a hangover and you can’t even remember the time. You can get out of bed at 1pm yet the time [for swallowing pills] has already passed (24-year-old PrEP user, non-adherer, and secondary school educated, single, mother of 1 and jobless).

That day I drunk and just came back and slept while it was still early. Then I slept and forgot to swallow them. I didn’t swallow, because by the time I woke up the time had passed already. I didn’t even bother to swallow it because the health worker told us that when the time passes that you are not supposed to swallow it and in fact I didn’t swallow it that day. (23-year-old PrEP user, adherer, secondary school educated, single, mother of 1, sex worker).

### Product attributes

Fear of the pill burden discouraged some of the participants from taking up PrEP, particularly given that they were not treating a particular disease. This was a common finding across all 26 participants. The quote below illustrates how this subject was discussed:

Nothing influenced me [to decline PrEP]. But let me say that I fear pills- I don’t like them. When I swallow them I feel bad. So, if I am not sick and I am just swallowing them, ah-ah [no] I cannot. (17-year-old non- PrEP user, secondary school educated, single and living with mother, considered emancipated minor because she works to cater for her and her mother’s livelihood).

Among the PrEP users, side effects resulting from using PrEP reportedly discouraged adherence. While many of the PrEP users experienced side effects and were able to overcome them, half of the PrEP non-adherers (n = 4) cited side effects as the reason for stopping. These four were all single and never married and were all older AGYW (18 + years). Two reported missing just a few days, one paused for a month and another had to stop completely because of the side effects, as reported by a 22-year-old and 24-year-old respectively:

Getting nausea in some moments. So I said that let me first give it [PrEP] a break for like a month and then I get back to using them. (PrEP user, non-adherer, primary school educated, single, and sex worker).

Everywhere I would scratch myself I would get swollen. So after 2 weeks I called the health worker and told her that I am having a bad experience with the drugs and then she told me to come to the health facility because I hadn’t yet even finished it [monthly PrEP prescription]. Then I went and I showed her and she said that “what are you thinking of doing” and I told her that I was thinking of stopping to use them [PrEP]. Then she told me that it is okay, you can stop using them (24-year-old PrEP user, non-adherer, secondary school educated, single, mother of 3 and jobless).

## Interpersonal factors

At interpersonal level, social support and fear of guardian’s reaction to PrEP use affected PrEP uptake and adherence among AGYW.

### Social Support

Some participants reported social support from friends, especially those who were also using PrEP and from family members to whom they had disclosed that they were using PrEP. Disclosure of PrEP use to sexual partners was not a common practice among participants because they feared that their sexual partners would suspect them of infidelity or that they would assume that they were HIV positive. The majority of the PrEP users (11/16) reported having disclosed PrEP use to either a friend or family member. Participants were influenced and encouraged to take up and adhere to PrEP by being reminded to take their pills or pick-up PrEP re-fills.

What influenced me was being at risk and also, my friends who came before me who told me about them [PrEP pills]. They told me that it is not bad and that they keep you safe especially us who are at risk. So I accepted and came and I also saw that I needed them. (19-year- old PrEP user, non-adherer, secondary school educated, single and mother of 2, and sex worker).

That sister of mine who I live with, she also reminds me. When I wake up she tells me that “You haven’t swallowed”. She reminds me and tells me that “first swallow before you go to work.” (24-year-old PrEP user, non-adherer, secondary school educated, single, mother of 1 and living with sister, cleaner).

### Fear of guardian’s reaction

This also seemed to play a big role in determining uptake of PrEP among younger participants who lived with guardians. The majority of the participants who declined PrEP lived with a guardian or relative (7/10). Out of those seven participants, three refused to take up PrEP because they feared being thought to be sexually active and feared that their guardian would assume that they were HIV positive. They were all mature and emancipated minors below the age of 17 and were single (never married). Two participants were quoted below expressing how fear of guardians discouraged them from taking up PrEP.

They say that it is a good drug. Even me I would also want to try but since I fear my mother, I am first waiting before I can get it (17-year-old non- PrEP user, secondary school educated, single and living with mother, considered emancipated minor because she works to cater for her and her mother’s livelihood)

The problem is that if she sees me using them, she may think that I went and had sex with a man who has HIV. And then I feared telling her. So it is very hard. (17-year-old non-PrEP user, primary school educated, single and living with grandmother, considered mature minor due to drug and alcohol dependence).

## Structural factors

### Access to health facility for PrEP refills

Travelling to distant areas and living far from the health facility made it difficult for some participants to pick-up PrEP re-fills. One 21-year-old who was unable to adhere due to travelling shared: “Remember that I missed a bit when my mother died. I went to the village for 2 months I think. And then I came back and began again.” Another non-adherent participant decided to stop picking PrEP from the clinic because the money she would spend on the transport fare was more than what was being reimbursed to her.

Now a boda [motorcycle] from that side up to here can cost around 7,000ugshs (approximately 2 USD). And remember that when you come here, by that time they were giving us transport of only 10,000ugshs (approximately 2.8 USD). So, I would just think about it-a boda of 7,000ugshs, then I come here and they give me 10,000ugshs and then I go back, you get it? It didn’t make sense because my sister used to live in Kasangati [approximately 18 km away from GHWP clinic] (20-year-old, secondary school educated).

A 23-year-old married mother of 1 who was enrolled as a good adherer at month 6 reported that she had stopped using PrEP during a follow-up interview during the month 12 follow up IDI. She shared the reason why she stopped using it: *“*I stopped using it [PrEP], because it is not available around here because I am very far.”

## Institutional factors

Adherence counselling, support tools and Incentives provided during study visits were all factors which facilitated adherence to PrEP.

### Adherence counselling from health care providers

Compelling educational messages and adherence counselling from health workers were factors that encouraged uptake of PrEP, and motivated participants to overcome challenges faced. Some participants reported that they had become discouraged about taking the pills, but resumed due to the counselling from health workers:

There is a time I even came here to tell the health worker that I am going to get off those pills. Because people think- even my sister who I live with, she had refused me from swallowing them and told me that “do you know that people are saying that you are sick of HIV”. Then I came here and told the health worker and she explained to me and told me to continue swallowing those pills and stop listening to people’s words because they will not save my life (23-year-old PrEP user, adherer, secondary school educated, single, mother of 1 sex worker).

Another participant who maintained good adherence throughout the study despite experiencing side effects shared one of the benefits of the counselling received:

I came and explained to the health workers my challenges [loss of appetite and stomach aches] and they told me to continue swallowing and if the condition doesn’t change in 3 months we can stop. But after 1 month of swallowing the condition changed and in the second month I didn’t have any challenges. The health workers are the ones who reassured me (Follow-up IDI, 23-year-old, good adherer until study exit at month 12, primary school educated, married, mother of 2, and jobless).

### Support tools

Devices such as phones, alarm clocks, watches and adherence assessment cards helped to keep participants on-track with their pill taking schedules and therefore aided them with adhering to PrEP. A 22-year-old adherer who works in a saloon spoke about her alarm: “I have an alarm. As soon as it clocks time I have to swallow the drugs.” Another participant shared the importance of adherence cards in the quote below,

That card [adherence assessment card given at the study site when a volunteer starts PrEP], let me say that it reminds me. Because I don’t want to see that space not filled. Whenever I see it I want to fill it and that is when I have swallowed it [PrEP]. In fact, whenever I finish swallowing, I take it out and tick it” (21-year-old PrEP user, adherer, secondary school educated, single, sex worker).

### Incentives

For some, the additional services and the benefits they got while on the study such as the reimbursements for study time and transport, free sanitary pads, free reproductive health services and treatment for common illnesses, continuous HIV testing, and education encouraged them to continue using PrEP until the end of the study. When asked what facilitated their adherence to PrEP, 2 participants spoke about how these extra benefits encouraged them to continue participating in the study and using PrEP.

The money [reimbursement for time] they give us is what helped me. Because when I leave here, I use the money which is given to me to buy food (Follow-up IDI, 23-year-old, good adherer throughout study, primary school educated, married and mother of 2, jobless).

Yes, I can’t stop using it [PrEP] because when we come here, the care that they give us and the way that they teach us, you keep updated about your health status. (Follow-up IDI, 24-year-old non-adherer at month 6, adherer at month 12, secondary school education, single, mother of 1 and jobless).

## Community factors

At community level; HIV related stigma, negative perceptions of PrEP in communities and social norms regarding sexual practices among AGYW affected uptake of PrEP. Participants also reported the impact of PrEP use on their sexual behaviour.

### Negative perceptions of PrEP in communities

Participants were asked about the community perceptions of PrEP. They reported hearing negative remarks about PrEP in communities or receiving negative reactions from people who they told about PrEP. Most of these were misconceptions and myths such as: No drug can prevent HIV since it has been around for long and that PrEP is Anti-Retroviral Therapy (ART) for infection and not for prevention. A participant reported people believing that PrEP is ineffective and that it is ART.

People [in the community] have their opinions. They say that there is no drug which can prevent HIV since it [HIV] has been here since a long time ago. They also say that the drugs are for people who are sick of [with] HIV. They say many things. (20-year-old PrEP user, non- adherer, tertiary level educated).

Other beliefs commonly heard in the community about PrEP were that PrEP users risk getting complications because of using it such as infertility and other diseases and that PrEP users are prostitutes, potentially attaching a stigmatizing identity to PrEP. A participant shared her experience:

We used to live with my sister and she told them about it [PrEP]. Now there was an older woman who said that “they are going to give those children drugs which are going to make them sick. Don’t allow them to go back”. (24-year-old Non-PrEP user, secondary level educated, single and currently living with mother).

### HIV related stigma

HIV related stigma discouraged PrEP uptake among some AGYW. It was variously reported that people in communities believed that PrEP was a form of HIV treatment and as such PrEP users were HIV positive. While many based their views on anticipated rather than experienced stigma, some participants reported actual experiences being labelled by friends or others in the community, as HIV positive, discouraging from continued use of PrEP:

When someone doesn’t know about those drugs, she or he can look at you when you are swallowing and they will wonder and say that this one may have gotten infected. So she can start to spread rumours about you. So even you sometimes you can say that instead of people talking about me, at least let me leave it [PrEP], I will not swallow it. (17-year-old non-PrEP user, primary school educated, single and living with grandmother, considered mature minor due to drug and alcohol dependence).

What has discouraged me, okay in the past when I had just started and then they [community] said those words [that I am HIV positive], then I said that let me leave these drugs. (24-year-old PrEP user, non-adherer, secondary school educated, single, mother of 1 and jobless).

Stigma was a common theme among participants. Adherers, non- adherers and those who declined PrEP reported fear of HIV stigma, as did married and unmarried participants. Those who turned down PrEP due to fear of HIV stigma (4/10) ranged from 17 to 24 years old.

### Social norms regarding sexual relationships and practices

Having multiple sexual partners was a common practice among this population, and participants also reported that their sexual partners likely did the same. Participants reported being prompted to take up PrEP because of the risk posed not only by their own multiple sexual relationships but also their partners’ multiple relationships. A participant whose adherence improved significantly with time described being worried by her sexual networks:

Sometimes you can have a man and when he also has like 3 other people and yet they also have other people. So that is what influenced me to say that let me start [PrEP]. (Follow-up IDI, 24-year-old non-adherer at month 6, adherer at month 12, secondary school educated, single, mother of 1 and jobless).

Though we did not collect detailed data from each participant on their sexual networks, we wish to note that it is common for women involved in sex work in Kampala to have regular partners whom they trust and are considered husbands/ boyfriends, alongside other casual partners/ clients.

While discussing the impact of using PrEP on norms regarding sexual behaviour, among both adherers and non-adherers, the most recurring concept was ‘feeling safe’. Describing the sense of safety that had resulted from using PrEP, AGYW frequently mentioned they felt comfortable; felt at peace; they had stopped worrying or become fearless. This suggests a renewed sense of security and confidence about being able to protect themselves from HIV, as illustrated by the following quotes.

I have become fearless knowing that since I am swallowing these drugs, I cannot get HIV. Because I do not trust my husband. So I have been fearless. (23-year-old PrEP user, adherer, primary school educated, married and mother of 2, jobless)

Yes, something has changed, because I see now that I have gained weight. I used to be so small (*laughs*). Now I am changing. I feel at peace because now I can protect myself. You never know I could have died if I hadn’t joined this study because I wasn’t keeping myself safe properly but now I am peaceful. (22-year-old PrEP user, non-adherer, secondary school educated, single and mother of 2, sex worker).

However, there were mixed views regarding the impact of PrEP on sexual behaviour of AGYW, with some saying they had noted a positive impact and others expressing concern about possible negative impact. For some PrEP users (5/16), the sense of safety or protection offered by PrEP led to an increase in risky behaviours, such as increase in the number of sexual partners and reduction in condom use relative to before using PrEP. These participants were mainly sex workers and entertainers (4/5), and all except one, had attained secondary education. These sex workers spoke about how some of them were now more inclined to take on more clients, including clients who were not willing to use condoms, because they knew that they had protection. For these AGYW, PrEP had given them a sense of freedom and control over decisions on their sexuality as shown by a 21-year-old adherer who identified as a sex worker: “For the men you just even increase the number if you are safe”.

Describing the impact on condom use, a participant reported:

I have reduced [condom use] a little bit because with my boyfriend we don’t use them so much, maybe the other one whom I don’t really know, I use them with him. It’s just that whenever I swallow PrEP I am comfortable (21-year-old PrEP user, adherer, secondary school educated, single, and sex worker).

Another 20-year-old participant reported that using PrEP had made her less vigilant to demand condom use with her clients:

It [PrEP] has helped me because even if someone [client] comes and tells me “I don’t want those things [condoms], I have money, let us have live sex” and yet I don’t know whether they are sick, I will just go. As long as I go and get checked. Because there is a day when I went with a man, I didn’t know but many people started telling me that he is sick [HIV positive]. Then I came back and they tested me and told me that I am negative. And then I even started liking it more and said that it means that they [PrEP pills] really work (adherer, secondary school educated, single and mother of 1, sex worker).

Even when some AGYW attempted to link increase in sex partners to other factors other than PrEP, in particular poverty, there was evidence of possible influence from the perceived protection offered by PrEP:

It is not PrEP that has made the men increase. For me it is poverty which brings them. When you are poor, anyone who comes, you can’t refuse. But if you don’t have protection, sometimes you can say that 5 [clients] are enough (24-year-old PrEP user, non-adherer, primary school educated, single, entertainer).

Those who reported a positive effect of PrEP on their sexual behaviour (7/16) stated that the experience of taking it had caused them to make a conscious decision to reduce risky relationships, to reduce risk of HIV and/ or to reduce the need to be dependent on PrEP to stay healthy. The larger part of this category of participants consisted of married and divorced/ separated AGYW (4/7), few in this group reported being involved in sex work and the entertainment sector (2/7). This category consisted of primary, secondary and tertiary level educated AGYW. These interviewees said they had to re-think their lifestyle and reduce number of sexual partners in light of the associated risks or due to the failure by partners to heed to HIV prevention and testing advice. For example, a participant described employing PrEP as a tool to end a relationship with a partner who declined to seek HIV testing:

[…] because for one of them when I showed him and told him that “this PrEP is the one which prevents HIV”, he told me that he cannot swallow that thing. And then I told him that “come and we go and get tested so that we can know where you stand” and he refused. So then I said that let me leave that one because I wasn’t sure about him and I remained with those 2 [sexual partners]. (24-year-old PrEP user, non-adherer secondary school educated, single and mother of 1, no job)

One of the main motivations for reducing partners appeared to be the need to demonstrate that they were transformed and saw value in preserving their health following PrEP, as demonstrated in the following dialogue:

#### Interviewer

Okay. What about the men who you have sex with, let me say their numbers- has the number of men increased or reduced ever since you started using PrEP?

#### Respondent

It has reduced.

#### Interviewer

What has caused you to reduce on the number of those men?

#### Respondent

I want to value my health now. Before I got PrEP, I had many boyfriends, they were 4. So now I have reduced them. For some of those who I wasn’t sure about their movements I reduced on them and remained with 2. (21-year-old PrEP user, adherer, secondary school educated, divorced and mother of 1, works in salon).

This perception appears to be confirmed by another participant who was using PrEP in the first place because of risky sexual behaviour, and rather than feeling safe and overconfident, she considered a change in lifestyle:

Just like how you can think to yourself that “you see I have gotten to the point of swallowing these drugs, let me reduce on the men so that I don’t get into problems”. Because you can find that if I didn’t have sex with them I would not get to the point of swallowing these pills. There is a way in which it has given me a lesson. (22-year-old PrEP user, non-adherer, primary school educated, single, sex worker).

There was a unique case of a participant whose inability to adhere consistently acted as an opportunity to completely change her sexual behaviour and stop sex work. She reasoned that the experience of being in and out of PrEP made her realize how much she remained at risk of HIV infection, and this prompted her to consider leaving sex work:

Oh, yes. I can say that it helped me because when I stopped using it, I decided and said that since I have stopped using PrEP which is what would have been protecting me and also considering that my man had told me that I would stay with him, I decided so that I could be able to keep myself safe. But it helped me in those moments. Because when I stopped using it I knew that I was going to be at risk and I would get it [HIV]. So I said that let me leave those things [sex work]. (Follow-up IDI, 24-year-old, non-adherer, secondary school educated, now married, mother of 3 and jobless).

## Discussion

In this qualitative methods study, we explore experiences with PrEP and factors influencing PrEP uptake and adherence in a cohort that offered daily oral PrEP to HIV high risk AGYW in an urban area in Uganda. Several determinants at all levels of the socio ecological model (SEM) were found to influence uptake and adherence behaviour in this population (Fig. 1). At individual level, reported low awareness of PrEP among AGYW prior to joining the clinical cohort confirms what has been reported in several quantitative methods studies carried out elsewhere [4, 19, 32, 33]. Studies have reported that low awareness is because most populations at high risk for HIV infection have not been given much consideration and therefore may have limited access to PrEP information [34–36]. However, in this study, some participants reported seeing or hearing the word PrEP in health facilities or through media but they did not pay any attention to it since it did not seem important to them. This suggests that despite the presence of some information on PrEP in communities, there is still not enough attention or interest in the subject. Uptake of preventive technologies tends to be problematic in many settings, and it could be postulated that AGYW involved in sex work might perceive no need for preventive measures if they believe they are HIV infected. However, we found that having a high HIV risk perception was one of the main factors that positively influenced uptake and motivated adherence to PrEP. In a study carried out among AGYW in Malawi exploring the association between HIV risk, risk perception and PrEP interest, it was found that while epidemiologic risk scores were positively associated with PrEP interest, high numbers of AGYW both above and below the high-risk cutoff were very interested in PrEP (68% vs. 63%)[37]. Factors contributing to AGYW believing that they were at high-risk of HIV infection included; involvement in sex work, having multiple sexual partners and mistrust of regular sexual partners, indeed some sex workers tend to have regular sexual partners alongside clients [38]. Similar findings have been reported among high risk populations in Uganda and Zimbabwe [39, 40].Our findings on barriers to PrEP uptake are consistent with other literature reporting that low HIV risk perception hinders PrEP uptake [33, 41, 42]. Alcohol use was also reported as a barrier to PrEP adherence and the same was reported in a study carried out among high risk women in Uganda [40]. Product attributes such as size of the drugs and associated side effects impeded uptake and adherence to PrEP as reported in another adolescent study [43].

At interpersonal level, uptake and adherence were affected by social interactions with guardians, family, and peers. Encouragement by close friends was also reported as a factor that motivated uptake of PrEP and AGYW in Kenya who were of a lower HIV risk profile (71% married and in monogamous relationships) compared to our higher risk study population [44]. Fear of guardian’s reactions were reported by some participants who declined PrEP. We found that some participants who lived with relatives or guardians found it easier to disclose PrEP use and received support while others found living with a guardian and fearing their reaction to PrEP was a barrier to uptake. In the group that feared to take up PrEP due to fear of their guardian’s reactions, participants were notably younger and were hesitant in taking up PrEP due to fear of being judged since younger girls were not expected to be sexually active. Other studies have also reported on the significance of a women’s’ age in their decision to take up PrEP and adhere to it [21, 22]. These findings highlight a challenge faced by AGYW and suggest that being younger may affect their willingness to take up and adhere to PrEP, likely due to the discomfort associated with disclosing their sexual activity and fear of being judged by parents/guardians, peers and other community members. There is need to explore how to better help younger at-risk AGYW to access PrEP in a way that protects their privacy from their guardians, peers, and the community at large. This also underscores the significance of paying attention to how environmental factors affect AGYW’s decision to take up PrEP and the importance of building social support systems for young PrEP users.

Our findings highlight the influence of community perceptions and stigma about PrEP use at community level. We found that there were misconceptions and mistrust of PrEP both in communities and among the AGYW themselves, with some believing that no medicine could prevent HIV since it is an incurable disease. Participants reported declining PrEP due to fear of stigma and fear of being judged by people in their communities. Studies with at-risk women and sex workers frequently show that stigma from the community significantly affects willingness to continue using PrEP [19, 45, 46]. Improving PrEP awareness is critical in efforts to increase PrEP uptake among AGYW in Uganda [47]. PrEP awareness campaigns should not only be geared towards widespread awareness but should target relevant messages to the needs of young people so as to capture their attention, as has been suggested in a study carried out among Men who have sex with Men (MSM) [48]. We also explored how PrEP use affected normative behaviour in this population regarding sexual practices such as having multiple sexual partners and condom use and we found that AGYW generally felt safe using PrEP, believing that they had protection against HIV. This appeared to have both positive and negative influences, with some increasing their risky sexual behaviour and others reducing it. This is consistent with findings from the United States among at-risk women in which PrEP reportedly made people less worried about HIV [33], and another among MSM which found that approximately half the participants adopted risk reduction strategies after starting PrEP, while the other half did not alter their behaviours [48].

Institutional level factors such as adherence counselling from health workers and support tools and incentives provided all served as facilitators to uptake and adherence to PrEP. Adherence counselling helped some participants to overcome barriers of side effects and HIV related stigma. Other studies have also highlighted the importance of adherence counselling in overcoming such barriers [23, 33]. Poor access to the health facility due to migration and living in distant locations acted as a barrier to uptake. Some participants missed refills or stopped using PrEP because they lived far and could not afford transport costs to the health facility. Accessibility concerns have also been reported among young people in Uganda and among a high-risk population in Zimbabwe [39, 43]. Unexpected migration among high HIV risk populations in Uganda has also been highlighted as a barrier to PrEP uptake and adherence [49].

Our study did have its limitations; firstly, we selected participants from a cohort study and findings may not be generalizable to at-risk AGYW who do not match our higher-risk enrolment criteria, or who decline to enrol in similar studies. Secondly, data was collected using interviews and responses are subject to social desirability bias. However, the interviewer was trained to encourage transparency and interviews were carried out in a private, quiet area to help participants to feel comfortable. Lastly, we did not have an objective measure of adherence. The study used pill counts and self-reports which are already routinely used in HIV treatment programs, but are prone to reporting bias. Adherence measurements using blood samples were done in one batch at the end of the cohort study, we were therefore unable to do real-time verification of reported adherence by those classified as adherers. The strength of this study is that we followed PrEP use over time, so we were able to explore real experiences of participants.

## Conclusion

There is need to address community misconceptions and increase awareness of PrEP to maximize PrEP uptake and adherence among AGYW. Targeted education messages should be developed to address misconceptions, doubts and fight stigma in communities and should be delivered in ways that attract the attention of AGYW. Counselling motivated adherence to PrEP and is an important aspect in PrEP programs. The socio-behavioural impact of PrEP use on sexual behaviour of AGYW needs to be studied further, both qualitatively and quantitatively.

## Data Availability

The datasets used and analysed during the current study are available from the corresponding author on reasonable request.

## Abbreviations

AGYW: Adolescents Girls and Young Women
ART: Anti-Retroviral Therapy
ARV: Anti-RetroViral
GHWP: Good Health for Women Project
HIV: Human immunodeficiency virus
IDI: In-Depth Interview
MSM: Men who have Sex with Men
IPAD: Interventions for HIV Prevention among Adolescents and Young Women
PrEP: Pre-Exposure Prophylaxis
SEM: Social Ecological Model
SSA: Sub-Saharan Africa
